# Fostering Resilience in Adolescents at Risk: Study protocol for a cluster randomized controlled trial within the resilience school-based intervention

**DOI:** 10.3389/fpsyg.2022.1066874

**Published:** 2023-01-23

**Authors:** Maria Llistosella, Clara Torné, Mercedes García-Ortiz, Griselda López-Hita, Ramona Ortiz, Laura Herández-Montero, Erika Guallart, Estefanía Uña-Solbas, Andrea Miranda-Mendizabal

**Affiliations:** ^1^Primary Health Care, Consorci Sanitari de Terrassa, Barcelona, Spain; ^2^Department of Nursing, International University of Catalonia, Barcelona, Spain; ^3^Primary Health Care, Institut Català de la Salut, Barcelona, Spain; ^4^Primary Health Care, Mútua Terrassa University Hospital, Barcelona, Catalonia, Spain; ^5^Department of Medicine, International University of Catalonia, Barcelona, Spain

**Keywords:** resilience, adolescents, health-promoting school, randomized controlled trial, mental health education

## Abstract

**Introduction:**

Resilience is considered of high relevance when developing interventions to cope with stressful situations. Schools are one of the key settings to promote resilience among adolescents. The purpose of this cluster randomized controlled trial is to assess the effectiveness of an intervention in adolescents at risk, aged 12-to-15 years old, to increase resilience and emotional regulation strategies.

**Methods:**

The recruitment period started in January 2022. Schools will be randomly allocated to control and intervention groups by an external researcher using computer-generated random numbers. The minimum sample size was estimated to be 70 participants per group. Primary health care nurses will carry out the intervention during the school period (January to June 2022). Students will follow a specific training consisting of six 55-min sessions, for 6 weeks. Each session will consist of 5 min of mindfulness, followed by 45 min of the corresponding activity: introducing resilience, self-esteem, emotional regulation strategies, social skills, problem-solving, community resources, social and peer support, and 5 min to explain the activity to do at home. Data will be collected at baseline, 6 weeks, and 24 weeks after the intervention. The child youth resilience measure-32 (CYRM-32) scale will be used to assess the effectiveness of the intervention. This study received a grant in June 2021.

**Discussion:**

The intervention is intended to improve mental health through resilience. Different factors related to resilience will be promoted, such as self-esteem, emotional regulation, social and communication skills, problem-solving and peer support, among others. As it has been designed as a cluster-randomized school-based intervention, we will directly ameliorate the participation and engagement of the target population. With the present intervention, we expect to improve coping skills in adolescents by enhancing resilience capacities.

## 1. Introduction

The incidence of mental health disorders in adolescents has become a global health burden (Kieling et al., 2011). The onset of most mental health problems is between 12 to 25 years old, with 20% affecting adolescents (Kessler et al., 2005; Wei et al., 2013).

During the COVID-19 pandemic, social inequalities widened and mental health problems in adolescents increased (Gracia et al., 2021; Hermosillo-de-la-Torre et al., 2021). Studies showed that during the pandemic, depression, anxiety symptoms, stress (Varma et al., 2021), and suicidal behavior (Gracia et al., 2021) increased among adolescents. Furthermore, several other risk factors affect the mental health of adolescents. Risk factors are those conditions related to the environment that increase the possibility of suffering poor adjustment or have negative effects on physical, mental, social health, or academic achievement (Braverman, 1999). The main risk factors identified in the literature are poverty or low socioeconomic level, maltreatment and sexual abuse, low-quality family environment, negative life events, and parents with mental disorders, among others (Llistosella et al., 2022). However, not all adolescents exposed to risk factors develop psychological distress. It is at this point that the phenomenon of resilience may appear.

Resilience is a phenomenon observed in adverse contexts, where risk factors can negatively impact psychological development (Wright et al., 2013). It is a dynamic and complex process (Masten, 2001) where many protective factors are involved. Protective factors can grow and develop over time, changing an individual capacity to face adversity and risk factors (Liebenberg, 2020).

Some models of resilience have been developed based on Bronfenbrenner (1981) and his theory of child development within a system of interactions between himself or herself and his or her immediate environment (family, social-wide: community and culture). These models organize in different domains diverse proximal and distal factors related to resilience that come from various environments (e.g., home, school, individual, etc.). They also describe the interactions between factors, domains, and individuals (Ungar, 2006; Llistosella et al., 2022).

For instance, Ungar’s resilience model, includes a new constructivist perspective, because they considered not only the internal aspects of the individuals and their interaction within different environment levels, but also, their cultural diversity which influenced individuals’ behaviors, beliefs, and relationships (Ungar, 2006; Ungar and Liebenberg, 2011). Most recently, we developed the Individual & Environmental Resilience Model (IERM) for adolescents and young adults (Llistosella et al., 2022). This model includes the main protective factors involved in resilience: relationships and social support, school engagement, presence of a positive mentor, family support, parental quality (e.g., housing, food, no violence), rules and routines, physical activity, coping, expressive skills, confidence, optimism, self-esteem, social skills and self-regulation (Llistosella et al., 2022). The IERM describes two major dimensions of resilience: (a) *Environmental*: including family, school, peers, cultural and community domains; and (b) *Individual skills:* including biological, behavior, communications, cognitive and emotional domains.

Resilience is related to positive mental health and it is also defined as good mental health and psychosocial functioning despite exposure to risk or adversity (Collishaw et al., 2016). Among children and adolescents, resilience encourages positive development, strengthens positive relationships with family and peers, increases success at work and school, improves coping mechanisms to adapt to adverse situations, and decreases the predisposition for anxiety, depression, and stress (Connor and Zhang, 2006; Masten et al., 2008). One specific resilience factor, the emotion regulation strategy (cognitive reappraisal and expressive suppression) included in self-regulation skill, has a positive impact on well-being, relationships, and social and psychological outcomes (Azpiazu Izaguirre et al., 2021; Yıldırım and Arslan, 2022).

Given the complexity of resilience, its promotion must be done with an appropriate intervention approach. Three different ways have been proposed: *Risk Reduction*, which focuses on reducing exposure to adversity; *Attentive to Assets*, which intends to increase the resources and their quality; and *Process-oriented*, which improves the life of the person at risk, instead of limiting susceptibility to risk or rising the number of resources (Masten et al., 2008).

For more than a decade, schools have been considered one of the key points to promote resilience and mental health (Greenberg, 2006). Several promoting-resilience training programs have been created and carried out in different contexts and populations, using different configurations, timings, and settings (Chmitorz et al., 2018), and have been proven effective (Suranata et al., 2020; Volanen et al., 2020; Zhang et al., 2021). Resilience interventions, based on cognitive-behavioral therapy (CBT; Dray et al., 2017) or, CBT with mindfulness techniques (Joyce et al., 2018), reduce depressive and anxiety symptoms, and positively influence individual resilience. Multicomponent interventions, for example, based on counseling or expert sports program, have shown effectiveness in increasing psychological resilience and reducing emotional disorders in adolescents with anxiety symptoms (Zhang et al., 2021). However, some other multicomponent interventions, have shown effectiveness in increasing only one of the resilience domains, but not as a whole (Kelley et al., 2021; Tripa et al., 2021). Therefore, there is a need to increase evidence on effective resilience interventions and especially those promoting mental well-being (Goldberg et al., 2019; European Commission, 2020).

Furthermore, most of the interventions previously done to promote resilience and mental health among adolescents have been conducted primarily by professionals other than nurses (Sugiyama et al., 2020; Kelley et al., 2021; Tripa et al., 2021). Primary healthcare is the first level of contact in the health system for individuals, families, and the community. The role of primary and community care nurses is a key piece for health promotion and prevention (Kemppainen et al., 2013). Nurses are strategically positioned to educate adolescents as active agents for their own health, to minimize risk behaviors, and promote positive lifestyle practices (Ahern et al., 2008). Adolescents should be at the centre of nursing practice, as well as their social and family environments. Indeed, nurses act as protective and facilitating actors for successful self-development and community skills for adolescents. It is fundamental to get them involved in the development, implementation, and assessment of interventions fostering resilience.

Based on the presented evidence and considering that about 70 to 80% of the population does not receive adequate care for mental health issues (Thornicroft, 2007), the promotion of resilience among adolescents could be appropriate for facing adversity in a stressful context such as the COVID-19 pandemic. Based on the IERM, the present protocol aims at describing a school-based resilience intervention to foster protective factors (individual, cultural, relational and community) in adolescents at risk, highlighting the role of the primary care nursing team as the main actor and driver of the intervention.

## 2. Study objectives and hypotheses

### 2.1. Objectives

- Evaluate the effectiveness of an intervention on resilience capacities in adolescents at risk aged 12-to-15
- Increase the emotional regulation strategies of adolescents at risk aged 12-to-15.
- Evaluate the association between resilience intervention and depressive symptoms in adolescents at risk aged 12-to-15 years.

### 2.2. Research hypothesis

We hypothesize that adolescents at risk between 12-to-15 years old, who receive a resilience school-based intervention, will increase their resilience capacities and emotional regulation strategies compared to the control group. Resilience will be associated with a decrease in depression symptoms in the adolescents who received the intervention compared to the control group.

## 3. Methods and analysis

### 3.1. Design

This study is a cluster-randomized controlled trial with parallel arms. Clusters will be school classrooms (grades 6, 7), subjects aged 12-to-15 years.

### 3.2. Participants

#### 3.2.1. Inclusion criteria

- Adolescents (girls and boys) aged 12-to-15 in a context of risk (risk of social exclusion or COVID-19 pandemic)
- Adolescents/parents who consent to participate in the study.

#### 3.2.2. Exclusion criteria

- Adolescents (girls and boys) aged 12-to-15 who do not want to participate in the activities of the intervention.

### 3.3. Study setting and data collection

Recruitment will begin after the start of the school year and the intervention will be carried out by the primary health care nurses throughout the school period (January/June 2022). Public or concerted (private schools which receive public funds or subsidies) schools in neighborhoods at risk of social exclusion from Terrassa, Manresa and Barcelona (Spain) will be invited to participate.

Before starting the intervention:

- An interdisciplinary committee of nine experts reviewed the intervention program and protocol in order to validate the contents and to ensure the relevance and appropriateness of the activities included in the intervention. The committee includes two psychologists, one social worker, and six primary health care and school nurses. Additionally, two adolescents at risk were included in the committee to develop the intervention and ensure the engagement of participants.

These nurses will be trained during two training sessions about resilience and activities included in the intervention.

- Researchers will contact the selected schools and will discuss the study with school directors and teachers, answering the questions and reviewing the consent forms. The representatives of the schools will sign the participation agreement.
- An online informative session for parents/carers will be held, where doubts will be solved.
- A letter with the informed consent and relevant information from the study will send to parents/carers by schools.
- Once the informed consent is returned from parents/carers, adolescents will receive a session with age-appropriate information about the study in their classroom conducted by their teachers and research team.
- Assent from the adolescents will be obtained.
- For adolescents who do not assent, each school will prepare another activity depending on each school. For example, readings sessions, time for studying or homework

**Figure 1:**
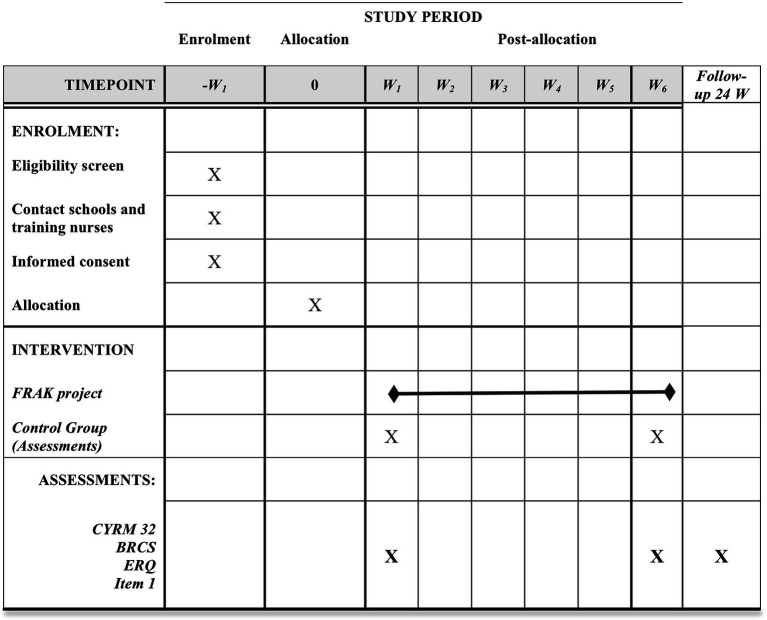
Fostering resilience in adolescents at risk study: Template enrolment, intervention and assessments. Adopting from figure SPIRIT. W, week; CYRM-32, child youth resilience measure-32; BRCS, brief resilience scale; Item 1, “Are You Depressed or Sad?”

**Figure 2:**
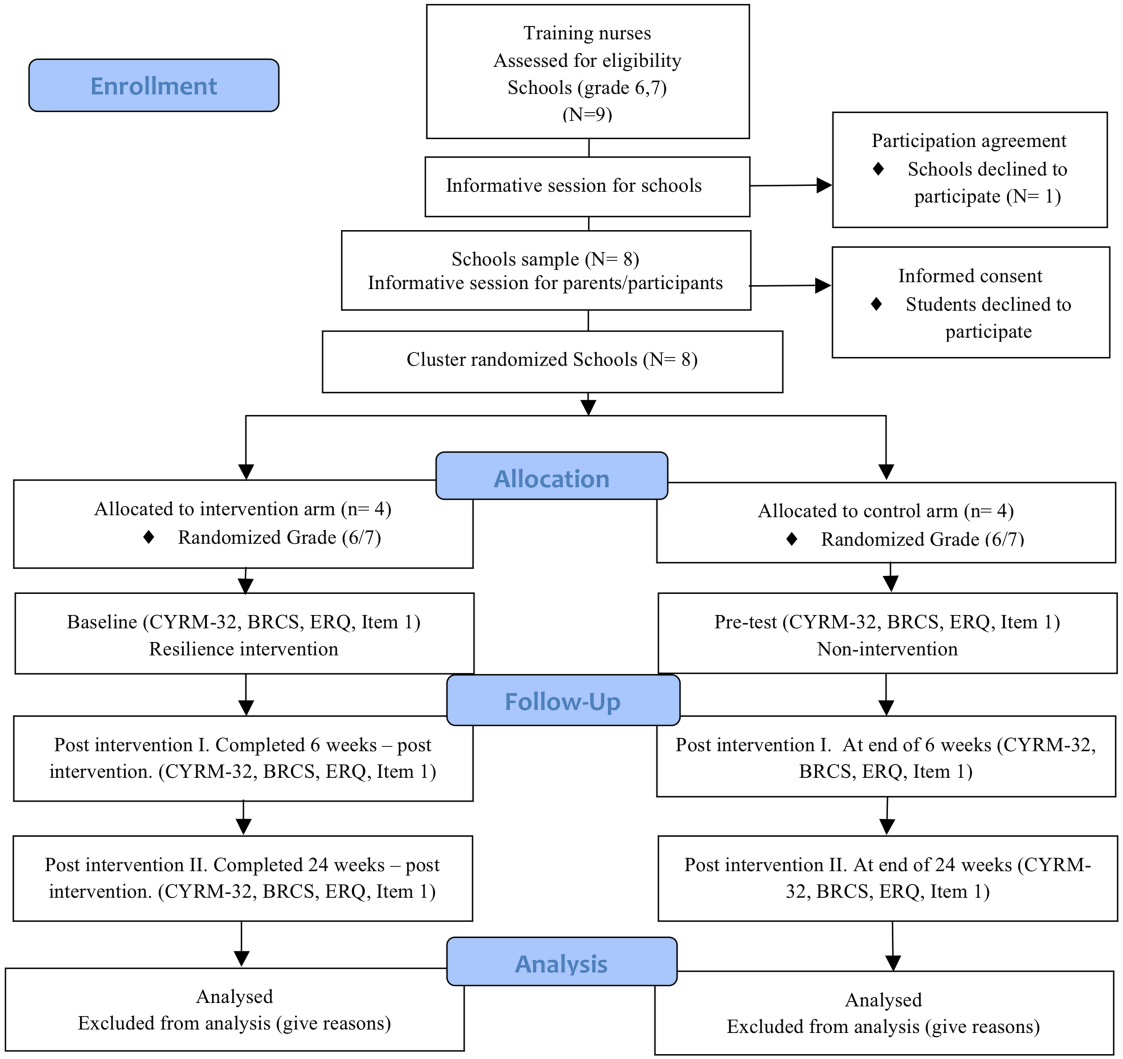
Consolidated standards of reporting clinical trial (CONSORT) flow chart, explaining the study settings, and how and when data will be collected. *N*, number of schools that have been involved in the specified step. CYRM-32, child youth resilience measure-32; BRCS, brief resilience scale; Item 1, “Are You Depressed or Sad?”

Data collection will start in January 2022 and will finish in December 2022. Data will be collected at baseline, after 6 weeks of the intervention (Post-intervention I) and 24 weeks later (Post-intervention II; Figure 1). Data from participants will be obtained through self-reported questionnaires provided by researchers. Teachers at schools will assure the full completion of the questionnaires at the desired times (T1-T3). Data will be stored on a secure server during the study, following the Spanish regulations. Recruitment of participants will be reported according to CONSORT guidelines for clustered randomized trials (Ivers et al., 2011; Figure 2).

### 3.4. Intervention

#### 3.4.1. Intervention group

The Fostering resilience in adolescent at risk (FRAK) intervention is a multicomponent intervention based on social and emotional learning, mindfulness and fostering protective factors. This intervention was developed according to the ecological framework, the empirical evidence and the contextual information. The contents, components, pedagogy, and technical elements of FRAK were established based on the IERM (Llistosella et al., 2022). Students will follow a training consisting of six 55-min sessions: (1) introducing resilience, (2) self-esteem, (3) emotional regulation strategies, (4) social skills, (5) problem-solving, (6) community resources, and social and peer support.

In order to decide which activities should be included in the final intervention we worked on three steps:

*First step*: The activities found in a previous systematic review were organized according to the protective factors with the greatest scientific evidence: resilience and coping; self-esteem; emotional regulation strategies, social skills, problem-solving, community resources, social and peer support, self-awareness, mindfulness, etc. (Llistosella et al., 2022).

*Second step*: A shared folder was created with all the material, to facilitate access to all members of the multidisciplinary expert committee. The mindfulness exercises were recorded on a podcast by a mindfulness expert and psychologist.

*Third step*: After two Delphi sessions, where the different activities were discussed and weighed, the intervention was designed.

The intervention group will receive six training sessions, each session will consist of 5 min of mindfulness, followed by 45 min of the corresponding activity and 5 min to explain the activity to do at home. All the activities included in the intervention will be dynamic and participatory; there will be no master classes. For this reason, the methodologies used will be real cases and role-playing. A complimentary reflective dossier was designed with individual activities to maintain the continuity of activities at home. More details about the sessions and their goals are presented in Table 1.

**Table 1:** Fostering resilience in adolescent at risk session’s outline.

Session goals	Activities
*Session 1:* **Introducing resilience and coping skills** Introduction to mindfulness. Introduction to conscious breathing Acquire mindfulness skills Learning the concept of resilience and coping	5-min Breathing space Describe the different protective factors according to the IREM model (individual and environmental factors). Recognize emotions through the body and strength resources. Put into perspective the impact of experiences of violence and discrimination during the course of their lives. The team resilience game: (1) participants from five groups; (2) Pick talent; (3) Write the talent on a name tag and put it on; (4) Each team one situation: Hurricane; Vandalism; Environment. How can your resilience team use their talents to cope with the situations? Reflexion and debriefing: What resilience skills are most useful in all situations? If you could add one resilience skill to your gear, what would it be and why? **Home practice:** Resilience Wheel: Identifying One’s Protective Factors
*Session 2:* **Self-esteem** Acquire mindfulness skills Increasing self-esteem and self-concept. Learning to value others positively and feel valued by them Expanding the repertoire of strategies to managestress Encouraging participants to bring strengths to the present to benefit their self-esteem and group integration.	5-min body scan, breathing space Development of the self-esteem definition and group reinforcement of self-esteem Express and recognize the qualities of peers: the peer in front will write a quality of the peer behind, then the one behind will communicate it and will write that of the next one and so on. Reflexion and debriefing: is there any model of family to develop good self-esteem? How many friends do I need to have good self-esteem?; What does school performance have to be like to have good self-esteem?; **Home practice:** Make an album with drawings, photos or any other resource with the themes: I live with…; What makes me feel better is…
*Session 3:* **Emotional strategies regulation** Acquire mindfulness skills Recognizing emotions in others and communicating one’s own emotions to others. Managing one’s own emotions and recognizing the emotion of anger and the resulting behavior	5-min body scan, breathing space Highway game and emotions: identify your unhelpful red thoughts (negative self-talk) and challenge them to come up with helpful green alternative thoughts (positive self-talk). Emotion check-in, psychoeducation about emotional regulation Reflexion and debriefing: what red useless thoughts make us feel sad, angry, and worried? what green thoughts make us feel happy, confident and brave? **Home practice:** Think of a situation that makes you very angry and try to capture it in an image
*Session 4:* **Social and communication skills** Acquire mindfulness skills Increasing knowledge of social skills. Learning the different styles of communication: aggressive, passive and assertive. Promoting assertive communication.	5-min body scan, breathing space A brief explanation of social skills and the three communication styles (passive, aggressive, assertive) Role-playing situations: “You meet a group of friends and there is a person you like a lot. He/she approaches you to talk and offers you a cigarette/alcohol, why he/she smokes or is consuming it. What would you do? Respond in an aggressive, passive, or assertive communication style. Reflexion and debriefing: how have you felt? how could we tell him to make him feel better? **Home practice:** Prepare your own “what would you do if…?.” To do this, prepare cards with situations to which it would be difficult for all of us to react (What would you do if… you have to do a job with someone you do not like?)
*Session 5:* **Solving problems, peers support and competence** Acquire mindfulness skills Promoting teamwork and personal competence. Increasing their ability to solve problems assertively and as a team. Learn to see the positive side of conflicts. Developing the capacity for reflection to solve conflicts. Promoting communication and dialog, to seek constructive solutions	5-min body scan, breathing space Life stories: different problematic situations identified by participants will be worked through the problem-solving technique (the participants work in teams to find solutions): (1) what is the problem? Define it, (2) brainstorm and list all possible solutions, (3) list what could happen for each solution, (4) select the best solution based on the consequences, (5) Make a plan for put the solution into practice and do it, (6) Evaluate the result in terms of strengths and weaknesses and, if it does not work, go back to step 2 and try again. Reflexion and debriefing: how have we resolved the conflict? **Home practice:** Resilience Wheel II: The purpose of the wheel is to identify one’s strengths and resources (Problems that I have solved, how I solve problems, that I can tell about myself…)
*Session 6:* **Community resources, social and peer support** Acquire mindfulness skills Learning about community resources Increasing community recourses	5-min body scan, breathing space Identify and learn the resources present in the community. Identify the possible barriers that can be found to access these resources and possible solutions to address them With the problems identified in session 5, the participants work in teams to find the different resources of the community to cope with these problems Closed activity: Make a mural on craft paper; large enough that all participants in 2–3 groups can contribute to the mural. They will be asked to represent on the mural what the group has meant to them, what they have learned, and their reflections. They will be provided with materials to make a group collage (draw, write) Guiding questions for the mural: how did you feel during these weeks? what do you feel you learned during the activities? what is the activity that you remember the most? what would you change or improve? what impacted you the most? what did they notice? **Home practice:** Resilience Wheel I: Identifying One’s Protective Factors

Eleven primary health care nurses will carry out the intervention. Ten of them are women with more than 10 years of professional experience with adolescents. The nurses will be trained by a resilience expert (principal researcher) during two formative sessions of one hour each, with the following content: (1) the concept of resilience and protective factors and (2) theoretical concepts and practical activities included in the intervention. In addition, the principal researcher will supervise the nurses, and meetings will be held weekly with the professionals during the intervention period. Nurses will carry out the intervention within their usual practice without receiving compensation or any other incentive to do it.

Students from four different schools will follow a 6-weeks training, consisting of weekly 55-min sessions.

To ensure adherence to the intervention of participants, primary health care nurses will carry out the six weekly sessions face-to-face during school hours with the teacher attending each of them. They will be done in groups of 25 to 30 students, depending on the school, but never more than 30 students, to facilitate the dynamics of the activities and participation.

#### 3.4.2. Control group

The control group will complete different activities depending on each school. As it has been stated: reading sessions, time for studying or homework, etc. They will fill in the same research questionnaires as the intervention group and during the same period (January to December 2022).

Schools in the control group will be all waitlisted to receive the intervention during the following academic year should if it proves to be effective.

#### 3.4.3. Monitoring procedure and risk participants

An independent monitoring committee will endorse the protocol and follow up on the process. This committee will meet regularly and evaluate the reports from researchers to assure and guarantee the rights and well-being of participants. The committee also will verify that the reported data are accurate and complete. The committee will be responsible for the overall supervision of the study. The following assessments will be conducted by the committee: (1) after the beginning of the study, (2) during the implementation of the intervention, (3) during follow-up, and (4) at the end of the study.

The research team agreed that in case of detecting a participant at clear risk (for example, depression), the nurses and the research team will inform the parents/carers. A list of public resources will be provided, and a visit will be suggested with the corresponding primary health pediatrician. The research team and nurses will follow up on the adolescent until the family contacts the referral pediatrician or during the study period. When necessary, the competent authorities in Social Services will be informed, to monitor the adolescent at risk. Confidentiality will be maintained to avoid the stigmatization of the participant.

### 3.5. Outcomes measures

#### 3.5.1. Primary outcomes

The level of resilience is the primary outcome, assessed using principally the Child youth resilience measure-32 (CYRM-32; Llistosella et al., 2019). Additionally, the Brief resilience scale (BRCS; Limonero et al., 2014) will be used.

##### 3.5.1.1. Child youth resilience measure-32

The CYRM-32 is a 5-point Likert scale, optimized to assess resilience in children and young adults (aged 12–23). It has been previously shown that Cronbach’s *α* for the scale was 0.88, Confirmatory factor analysis supported a three-factor solution: family interaction (0.79 Cronbach’s *α*), interaction with others (0.72 Cronbach’s *α*) and individual skills (0.78 Cronbach’s *α*). Pearson correlation to assess temporal stability over 2 months was 0.695 (Llistosella et al., 2019).

##### 3.5.1.2. Brief resilience scale

The BRCS scale (Limonero et al., 2014; 4-items) is also a 5-point Likert scale to assess coping strategies to manage stress. Cronbach’s *α* for the scale was 0.7. Temporal stability at 6 weeks was measured by Pearson’s correlation, and its value was 0.69.

#### 3.5.2. Secondary outcomes

As secondary outcomes, we will evaluate the emotional regulation strategies through The Emotion Regulation Questionnaire (ERQ; Gross and John, 2003), and sadness symptoms through the assessment of a single item *“Are you depressed or sad?”* (Chochinov et al., 1997).

##### 3.5.2.1. Emotion regulation questionnaire

The ERQ will be used to assess two emotional regulation strategies: cognitive reappraisal (6 items) and expressive suppression (4 items). Participants will report using a 7-point Likert scale (1 = strongly disagree, 7 = strongly agree). Cronbach’s *α* coefficients were 0.75 (Suppression) and 0.79 (Reappraisal). Pearson coefficient was used to evaluate test–retest reliability over 3 months, and the values were 0.66 (Suppression) and 0.64 (Reappraisal; Gross and John, 2003).

##### 3.5.2.2. Depressive symptoms

Depressive mood will be asked as follow: *“Are you depressed or sad?.”* It was adapted from the “Are you depressed?” Screening for depression in the terminally ill (Chochinov et al., 1997). It evaluates through a numerical scale of 0–10 (0-not depressed, 10-worst possible depression).

##### 3.5.2.3. Sociodemographic variables

Adolescents will be asked to self-report sociodemographic variables: age, gender, ethnicity and family structure.

### 3.6. Sample size calculation

Standard deviation (SD) of the primary outcome CYRM-32 scale was used to estimate the minimum sample required to detect a 10-point difference in the scale between the intervention and control groups.

Taking into account an SD of 20 of the CYRM-32 scale, accepting an alpha risk of 5%, power of 80% and a follow-up loss rate of 10%, it would be necessary a minimum of 70 students/group. GRANMO tool (Antaviana, 2011) was used to calculate the sample size.

### 3.7. Randomization

Eligible schools will be randomly assigned to the intervention or control group. The clusters will be school classrooms (grades 6 and 7). For each grade (6 and 7) there will be several classrooms (clusters) depending on the schools. The randomization procedure will be performed in two steps. First, the schools will be randomly allocated either to an intervention group or control group by the external researcher using computer-generated random numbers; second, the external researcher will randomize the intervention group into the different school levels.

According to the minimum estimated sample size (70 participants/group) and knowing that there are 25 to 30 students per classroom, we will need a minimum of three clusters (classrooms) per group.

### 3.8. Blinding

Given the difficulty in masking any condition, participant groups and personnel (research team) will not be blinded.

### 3.9. Data analysis

An intention-to-treat analysis will be performed. A descriptive analysis of the variables will be carried out, with frequency and percentage for the qualitative variables; mean and standard deviation for the quantitative normal distributed variables and median and quartiles for the quantitative not-normal distributed variables. The values from the scores for the intervention and the control group will be compared with the *t*-student test if the score is normally distributed, with the Mann–Whitney test if the score is not-normally distributed and if the score has a cut-off, it will be compared with chi-square test. The effect size will be calculated as the difference of means and with ratio of proportions (with its corresponding confidence intervals).

A linear regression and a logistic regression will be considered including other variables that can affect the response. Also, we will stratify the results for other factors like gender, age and others than will be considered. To check normality, the Kolmogorov–Smirnov test and the Shapiro–Wilk test will be used. All the confidence intervals will be performed with a 95% confidence level. All the analysis will be performed with the software R version 4.2.2.

## 4. Discussion

The present study describes the design, implementation and assessment of the effectiveness of an intervention based on the Individual & Environmental Resilience Model (Llistosella et al., 2022) aiming to increase resilience in adolescents at risk. Moreover, the intervention aims to reinforce the resources and protective factors of adolescents, especially self-regulation skills.

Self-regulation skill as an emotional regulation strategy contributes to the resilience process and also has a positive impact on well-being, relationships, and social and psychological outcomes (Azpiazu Izaguirre et al., 2021; Yıldırım and Arslan, 2022).

According to Gross’ model, there are two main strategies for emotional regulation: cognitive reappraisal and emotional suppression (Gross and Thompson, 2007). The first consists in reframing or re-interpreting the meaning of an event in order to experience it more appropriately, reducing negative behavioral and physiological responses (Gross, 2002). The mechanism of the second one is based on the decrease in emotional expression that aims to hide the emotions that individuals are experiencing (Gross and Thompson, 2007). Studies have shown that these strategies often differ in emotional effects and adaptive psychological adjustment. They could be presented either as adaptive strategies or maladaptive strategies, depending on the expected emotional, behavioral, and cognitive outcomes (Aldao and Nolen-Hoeksema, 2012).

The present study is very powerful because: (1) the content validity of the intervention was determined by nine experts and two adolescents at risk from the multidisciplinary expert committee; (2) it will promote different factors related to resilience, such as emotional regulation strategies, social and communication skills, problem-solving and peer support, among others; all of them have been related with resilience and described in the literature (Dray et al., 2017; Gartland et al., 2019; Pinto et al., 2021; Llistosella et al., 2022); (3) it is dealing with adolescence, a period when several biological and psychological factors can affect and modify the normal development of the individual (Azpiazu Izaguirre et al., 2021). It is also a period to lay the foundation for well-being and foster positive development, including resilience (Morrish et al., 2018). Furthermore, resilience-based interventions have been proven effective only among adolescents between 12-to-18 years and not among children under 12 years; (4) It will assess resilience pre and post-intervention, and also at 24 weeks ensuring the assessment of mid and long-term effects of the intervention; (5) a control group will be used to ensure that the effect is due to the proposed intervention; (6) it has been designed as a cluster-randomized school-based intervention to facilitate adolescents participation and engagement; (7) the static analysis plan was developed by a statistical analysis expert from the International University of Catalonia; and finally (8) an expert also reviewed the final version of the protocol. And the intervention has been drafted to be carried out in more than one school, training and tutoring the team to give reliability and stability to the protocol. Recommendations for Interventional Trials (SPIRIT; Guidance for Clinical Trials Protocols, 2021.) were used to develop the *Fostering Resilience Adolescents at Risk* protocol. Additionally, the CONSORT 2010 Checklist was reported in additional file 1.

In the application of this intervention, we consider some limitations. On one hand, the effect of the intervention may be lost in the long term. However, a 24 weeks follow-up is proposed to assess the maintenance of the effect and see if it is necessary to include reinforcement or repeat the intervention after 1 or 2 years to maintain resilient results. On the other hand, there may be some disparities in the effectiveness of the intervention between schools due to the different teams involved. If this is the case, we will propose to improve the trainings so that the teams act as similarly as possible.

Once the intervention will be applied, we will be able to assess its effectiveness. We expect to increase resilience in adolescents at risk and prevent mental health problems through this school-based intervention. Once the effectiveness is demonstrated, nurses from different fields such as primary health care or mental health specialists will have access to the program to be implemented in schools or other sites that work with adolescents at risk. This intervention will give new tools to nursing professionals to increase resilience and coping skills for facing adverse situations during adolescence.

Currently, we have finished the recruiting period and the trial status is “active” at www.clinicaltrials.gov. At the end of the recruitment, we had a total sample of 674 participants from eight randomized schools. The intervention was carried out in 4 schools from January 2022 to spring 2022. We are analyzing the initial data from control and intervention groups at T1 (baseline) and T2.

## 5. Ethical considerations

### 5.1. Approval

The present protocol was reviewed and approved by the Ethics Committee Institutional Review Board of the “Consorci Sanitari de Terrassa” (Ref: 02-21-160-016) on July 26, 2021.

### 5.2. Consent process

The study will be carried out in compliance with current legislation, by following the Biomedical Research Law 14/2007. The confidentiality of participants’ data will be guaranteed, in compliance with Organic Law 3/2018, of 5 December, on the Protection of Personal Data and the guarantee of digital rights and the General Data Protection Regulation (EU) 679/2016.

First, secondary school directors who will participate in this research will sign an agreement and attachment to the study. Second, an online session with parents/carers of the selected schools will be held to explain the study and the intervention. Third, before being registered for the study, the schools will send a letter with all relevant information about the study and the written informed consent form (in both languages, Catalan and Spanish, to parents/carers). Parents/carers will return the informed consent form (ICF) signed and they will keep a copy. ICF from participants will be obtained after an informative session (with age-appropriate information) in the classroom conducted by their teachers and the research team. If the parents/carers give consent, but the adolescents do not want to participate in the study, the decision of the participants will prevail and will be respected. For adolescents with difficulty with written information, visual information sheets will be provided. Each participant will be identified by a serial number and the initials of the first and last name. Only the principal investigator and collaborating researchers will have access to the collected data.

## Ethics statement

The present protocol was reviewed and approved by the Ethics Committee Institutional Review Board of the “Consorci Sanitari de Terrassa” (Ref: 02-21-160-016) on July 26, 2021.

## Author contributions

ML: conceptualization, methodology, supervision, and writing – original draft preparation. CT, MG-O, GL-H, RO, LH-M, EG, EU-S, and AM-M: conceptualization and writing – reviewing. All authors contributed to the article and approved the submitted version.

## Funding

This study was funded by a grant from the Strategic Plan for Research and Innovation in Health from the Departament de Salut de la Generalitat de Catalunya (Spain; SLT017/20/000070).

## Conflict of interest

The authors declare that the research was conducted in the absence of any commercial or financial relationships that could be construed as a potential conflict of interest.

## Publisher’s note

All claims expressed in this article are solely those of the authors and do not necessarily represent those of their affiliated organizations, or those of the publisher, the editors and the reviewers. Any product that may be evaluated in this article, or claim that may be made by its manufacturer, is not guaranteed or endorsed by the publisher.
